# Staff expectations for the implementation of an electronic health record system: a qualitative study using normalisation process theory

**DOI:** 10.1186/s12911-019-0952-3

**Published:** 2019-11-14

**Authors:** Carolyn McCrorie, Jonathan Benn, Owen Ashby Johnson, Arabella Scantlebury

**Affiliations:** 1 Patient Safety Translational Research Centre, Bradford Institute of Health Research, Bradford Royal Infirmary, Duckworth Lane, Bradford, BD9 6RJ UK; 2 School of Psychology, Faculty of Medicine and Health, University of Leeds, Leeds, LS2 9JT UK; 3 School of Computing, University of Leeds, Leeds, LS2 9JT UK; 4 York Trials Unit, Department of Health Sciences, ARRC Building, University of York, York, YO10 5DD UK

**Keywords:** Electronic health records, Implementation, Patient safety, Normalisation process theory

## Abstract

**Background:**

Global evidence suggests a range of benefits for introducing electronic health record (EHR) systems to improve patient care. However, implementing EHR within healthcare organisations is complex and, in the United Kingdom (UK), uptake has been slow. More research is needed to explore factors influencing successful implementation. This study explored staff expectations for change and outcome following procurement of a commercial EHR system by a large academic acute NHS hospital in the UK.

**Methods:**

Qualitative interviews were conducted with 14 members of hospital staff who represented a variety of user groups across different specialities within the hospital. The four components of Normalisation Process Theory (Coherence, Cognitive participation, Collective action and Reflexive monitoring) provided a theoretical framework to interpret and report study findings.

**Results:**

Health professionals had a common understanding for the rationale for EHR implementation (Coherence). There was variation in willingness to engage with and invest time into EHR (Cognitive participation) at an individual, professional and organisational level. Collective action (whether staff feel able to use the EHR) was influenced by context and perceived user-involvement in EHR design and planning of the implementation strategy. When appraising EHR (Reflexive monitoring), staff anticipated short and long-term benefits. Staff perceived that quality and safety of patient care would be improved with EHR implementation, but that these benefits may not be immediate. Some staff perceived that use of the system may negatively impact patient care. The findings indicate that preparedness for EHR use could mitigate perceived threats to the quality and safety of care.

**Conclusions:**

Health professionals looked forward to reaping the benefits from EHR use. Variations in level of engagement suggest early components of the implementation strategy were effective, and that more work was needed to involve users in preparing them for use. A clearer understanding as to how staff groups and services differentially interact with the EHR as they go about their daily work was required. The findings may inform other hospitals and healthcare systems on actions that can be taken prior to EHR implementation to reduce concerns for quality and safety of patient care and improve the chance of successful implementation.

## Background

Electronic health records (EHR) - digital, longitudinal records of patient’s health and healthcare that can be shared by different healthcare professionals [1] - are being introduced into many healthcare organisations around the world [2]. Global evidence exists for the potential impact of EHR implementations to improve record quality, increase administration efficiency, and support better quality, safety and coordination of care [1, 3, 4]. However, there is growing recognition that implementing an EHR across complex hospital care systems remains a major challenge world-wide [1, 5–10], with it estimated that more than half of all systems fail, or fail to be properly utilised [11]. Progress in EHR implementation in the United Kingdom (UK) secondary care hospitals has been particularly slow [11–15], with previous negative experiences contributing to a disengaged workforce.

In 2002, the UK Government made a significant financial commitment to EHR implementation with its National Programme for Information Technology in the National Health Service (NPfIT) [16]. The programme was widely criticised for its failure to implement EHRs in UK secondary care hospitals [17]. This was despite previous local initiatives achieving nearly 100% coverage with lifelong EHR in primary care [18]. The slow progress in UK hospitals was largely due to the challenges of integrating relatively inflexible procured software systems into National Health Service (NHS) organisations in which local needs vary – or are locally perceived to vary [11]. Since NPfIT, the UK Department of Health has produced several, costly initiatives to achieve its goal of an integrated digital care record within hospitals. For example, ‘Safer hospitals, Safer wards: achieving an integrated digital care record’ [19], is estimated to have cost the NHS £500 million since its launch in 2013, with £60 million of the first instalment being unallocated due to local NHS trusts failure to demonstrate a return on investment. In 2015, NHS England committed a further £100 million to support delivery of EHRs [20]. To promote the new ambition for a paperless NHS by 2023 [13], the Health Secretary has committed £4.2 billion over the next 5 years [21], which highlighted the UK Governments’ conviction that investing in digital technology will improve NHS care delivery.

Progress in the UK is confounded in part, by a lack of evidence on the associated challenges of EHR implementation, with the majority of evidence originating from North America; presumably due to the maturity of systems there, as compared with health organisations in other parts of the world [22–30]. A review of realised benefits [31] identified only five UK-based studies [32–36], with very little evidence reporting perceived improvements in care quality and safety. Instead, benefits were mainly related to availability and accessibility of information. These findings reflect the focus of the studies which were mainly concerned with the general impact of implementation both before and during initial implementation. In the UK, the NHS is centrally funded and has national strategies that are adopted at local level. Given the major differences in the social, political and economic foundations of the UK health and social care system, it is important to explore whether the benefits derived from EHR are relevant to other contexts.

There is a limited, but emerging evidence base on aspects of context that play out as barriers and facilitators to digital health interventions [37, 38]. The Medical Research Council’s framework for developing and evaluating complex interventions states that taking account of context is crucial to implementation [39]. Most effort in preparing for EHR implementation are directed towards organisational readiness, including staff readiness [40]. A recent review reported that for 95% of included studies, users were the primary barrier to implementation [41], and an additional review highlighted the importance of human factors in the success of, and barriers to implementation [42]. Greenhalgh and colleagues summarised the evidence as to why this might be case and concluded that “it is not individual factors that make or break a technology implementation effort but the dynamic interaction between them” ( [43], p.2).

Many different approaches have been used in order to produce rich theorisations of implementation of technology-supported health and social care initiatives, but these academic outputs have not been directly accessible to front-line hospital staff. In an effort to plug this gap, several studies have drawn on the literature to produce frameworks to inform implementation [43, 44]. To ensure successful implementation and avoid further financial waste, we need to understand better how users of EHR can be facilitated through implementation, pay more attention to factors associated with disengagement and translate this understanding across whole healthcare service organisations.

## Methods

### Aim

The aim of this study was to explore health professionals’ expectations of change and outcome following procurement of an EHR. In particular, we were interested in testing the relevance of Normalisation Process Theory (NPT) [37, 45–47] as a framework to understand perceived implementation factors prior to single healthcare organisation-wide EHR implementation.

### Study design

A theoretically informed, qualitative exploratory study was conducted to explore health professionals’ expectations of EHR implementation at the study site. Interviews were semi structured and were conducted during November 2016.

### Theoretical approach

A review of implementation of e-health systems concluded that most of the literature is focused on organisational issues, neglecting the wider social framework that must be considered when introducing new technologies [48]. Rather than focusing on predictors of behaviour, Normalisation Process Theory (NPT) focuses on the work that individuals and groups do to integrate interventions into routine practice [37, 45–47]. It can help in understanding why some processes seem to lead to a practice becoming normalised while others do not. NPT proposes that implementing technology can be achieved through ‘energising’ four mechanisms: Coherence (understanding of reasons for implementation and potential value of the technology), Cognitive participation (preparedness to engage and commit to use the technology), Collective action (ability to do the work to use the technology) and Reflexive monitoring (how staff appraise the technology) [45, 47, 49]. It is generally accepted that NPT provides a consistent framework that can be used to describe, assess and enhance implementation potential [49–57]. The mechanisms have high stability across settings and, notwithstanding challenges in applying NPT in terms of managing overlaps between constructs, there is evidence that it is a beneficial heuristic device to explain and guide implementation processes [58].

Recently, NPT has been used to explore implementation of digital health interventions [59, 60], including introducing EHRs in specific care settings [49, 61]. As far as we are aware, no studies have used NPT to explore users’ expectations of change and outcome following procurement of an EHR that will be implemented across an entire healthcare organisation. Using NPT to explore health professionals’ expectations could generate a better understanding of how they can best be facilitated through the adoption process. This understanding is vital for those managing the change process.

### Study setting

The study site was an NHS teaching hospital trust in the North of England. In November 2016, when the study took place, the hospital served a population of over half a million people. Annually there were around 135,000 attendances at Accident & Emergency, 121,000 in-patients, and around half a million out-patients. The population had increased by over 10% in the last 10 years, and was predicted to have increased by almost a third over the next 15 years.

There were approximately 6000 people employed by the Trust of which around 5000 would be expected to use EHR during the course of their daily practice. It was estimated that approximately two-thirds of the workforce had little or no experience of using EHR prior to their engagement with the local implementation strategy. A great deal of the patient record work of the Trust was completed on paper. At least 44 clinical systems and clinical data repositories were being used in distinct areas within the Trust and previous attempts at introducing a version of an EHR or systems that had some of functionality of an EHR had not been joined up. To remedy this, the Trust developed a strategy for implementation of a single, trust-wide EHR system.

### Description of preparing for EHR use

A local, comprehensive procurement assurance process commenced in March 2015. The EHR system came 80% preconfigured with the expectation that the remaining 20% of the functionality of the EHR could be adjusted to meet local requirements. The strategy to prepare EHR users for their expected use of the system had a number of components. These included clinically-led development of the strategy implementation plan; strategy “road shows” across clinical areas which included raising awareness of the EHR and its potential benefits to staff, as well as allowing staff to identify areas of concern; formal on site demonstrations and training; additional interactive demonstrations and e-learning materials including an online ‘play-domain’ (a dummy version of the system that staff could use to practice after training) and; additional training to staff designated as ‘EHR friends’ or ‘super-users’ to support the ‘go-live’ weekend.

In the original plan, the Trust was planning to go live in November 2016. The implementation phase lasted until the immediate preparation for the go-live weekend, which happened in September 2017. Our study was conducted during November 2016, prior to an official announcement of a decision to delay going live, and as such this overview of the implementation phase is concerned with strategies that were used prior to the original go live date.

### Sampling and recruitment

Data was collected from health professionals and members of the EHR implementation team. A purposive sampling frame was used to recruit hospital staff who represented a range of staff groups and grades. Participants were recruited from four hospital departments, which were considered to broadly represent the key services provided by the hospital, namely: Accident and Emergency (A&E), General Surgery, Rheumatology-outpatients and Elderly care (in-patient). Permission was sought from department managers and/or senior staff in each service to recruit staff to interview. Recruitment emails were then sent either by the senior member of staff and/or qualitative researcher and these asked staff to contact the research team directly should they wish to take part. Some snowball sampling was also used to recruit participants, either by asking health professionals to recommend colleagues at the end of each interview, or by the researcher being approached directly whilst visiting the wards for data collection.

### Participants

Fourteen members of staff who represented a variety of staff groups (7 doctors and 5 nurses of different grades, a hospital manager and a ward clerk) and departments (Accident and Emergency, Assessment, Outpatients, General Medical, Anaesthesia, Neurology and Stroke) within the study site took part in semi-structured interviews. Three interviewees were involved in the EHR implementation project, as members of the support team or as super-users.

### Interview design and content

Qualitative, face-to-face, semi-structured interviews were conducted and lasted between 11 and 40 min. The majority of participants were interviewed individually with three participants interviewed together. A topic guide (Additional file 1) provided the framework for the interviews, and was informed by the Normalisation Measure Development Questionnaire (NoMAD) instrument, and NPT implementation toolkit [47, 62–64]. Participants were asked about their perceptions of the benefits and barriers to implementation of EHR, as well as the perceived impact of the system upon their practice and patient safety.

### Analysis

With consent from participants, all interviews were audio-recorded and transcribed verbatim. Analysis was facilitated by use of the qualitative data management programme NVIVO 12. CM conducted the analysis, with regular discussions held with JB and AS during coding and theme development. Analysis was conducted in two stages. The first involved initially analysing all data thematically, following guidance as outlined by Braun and Clarke [65]. Theme and sub-theme development was largely deductive, using a-priori codes dictated by interview questions (e.g. perceived impact on participants’ existing work practices), whilst allowing for emergent themes. The second stage of the analysis involved mapping themes from the initial thematic analysis onto the four core mechanisms of NPT. This facilitated understanding of participants’ expectations of the EHR; their understanding of why it was being implemented; their engagement with and commitment to implementation and their perceptions of the impact, benefits, barriers and disadvantages of implementation. The outcome of the analysis was a researcher commentary on emergent theory with supporting quotes.

## Results

The four core mechanisms of NPT provided a structure for interpreting the findings. A summary of the themes and sub-themes is shown in Table 1 below. It is important to consider that activities in all four domains may occur concurrently, and relations between these core concepts are not linear. Even so, they focus attention down on “*how the work gets done”* [46]. An overview of the coding framework is shown in Additional file 2.

**Table 1:** Summary of themes and sub-themes

Theme	Sub-theme
Coherence	Understanding of reasons for introduction
	Purpose of EHR
	Anticipated benefits
	Who think will benefit
	How it differs or compares to paper records
Cognitive participation	Concerns that have about using the system
	Training and support
Collective action	Perceived impact on practice
	Perceived impact on existing work practices
	Perceived impact on working relationships
Reflexive monitoring	Perceived long-term benefits
	Perceived opportunities to adapt system
	Perceived barriers to use
	Disadvantages to use

### Coherence – do staff understand the reasons for EHR implementation and the potential value of incorporating use of the EHR into their routine work?

The extent to which health professionals understood the value in implementing EHR was strong amongst participants, with digitisation seen as a normal part of working in the modern NHS. Despite universal acceptance of the potential value of the EHR, staff groups varied in their perceptions of the intended purpose of the EHR. For nurses and ward clerks, the purpose of the EHR was perceived as data-centric (data storage and sharing):

*Nurse (Sister): “It’s going to become more of a task-orientated job, where you’re having to input stuff into EPR, rather than just getting on and carrying on with your clinical work like you normally would … ” (10:54-57).*

Clinicians, on the other hand, viewed EHR as treatment-centric and an aid to patient flow and decision making:

*Doctor (Consultant): “ … you will be able to get a better overview of the department, so to run the department will probably be easier.” (3:289-290), and Manager: “[It will] reduce clinical variation, improve the safety of care for patients and drive decision-making … it tells them [clinicians] what to do so we get consistency in practice.” (7:181-185*).

The idea of using an EHR in routine practice was strongly supported by the majority of participants, with a number of anticipated benefits proposed. Participants were particularly enthusiastic about the prospect of having all information in one place. The majority of views coincided with the ‘official perspective’ of the anticipated rewards of EHR implementation. The presentation of information in a standardised, legible format was particularly well-received:

*Doctor: “The thing that I am looking forward to most is being able to read the consultant’s writing, which I, personally, struggle with at the moment, whereas if it’s dictated and typed there is no, sort of, room for error. So, that is the best part of it for me.” (2:114-116).*

Use of the EHR was expected to improve efficiency of transfer of information between different specialties, leading to improved prescribing and test requests: “*… the electronic prescribing, the electronic requesting, those things will be better”. (Consultant, 4:558–559).* One participant, with direct responsibility for hospital governance, expected that over time, use of the EHR would improve capacity for audit and research. This would optimise opportunities to produce robust evidence for good quality care, as well as highlighting areas for improvement:

*Manager: “ … once it settles in … the benefits of what the outputs are from the system … I think it will prove that actually we deliver high quality care across the board. And then we’ll know the areas where we don’t and we can target them.” (7:161-173).*

Beyond improving access to and legibility of information, the anticipated benefits of the EHR varied across and between staff groups and services. EHR implementation was expected to be of most benefit to the working practice of junior doctors. For example, it was expected that the risks of missing important information or steps required within clinical decision making processes would be minimised through prompts to enact specific protocols within the EHR:

*Doctor: “ … when you try to do a ward round for a person, or clerk somebody in, you physically can’t do anything until you do a VT prophylaxis, until you put their weight to prescribe a drug … if you prescribe a blood thinning medication … somewhere it forces you to do a certain score of their risk of bleeding … things that basically can be missed out quite often if we are doing paper versions.” (2:138-144).*

It was less clear how nurses would benefit, particularly with regards to the volume of information that they would need to record into the system. Nurses were concerned as to how important information could be safely passed on to their colleagues:

*Nurse: “ … we don’t physically know how we are going to give handover … people worry about how that’s going to happen safely, for the information to be passed on safely from one shift to the next … because there’s a lot going on, tests and results chasing, all that sort of thing … ” (1:47-54).*

Nurses were also concerned that using the EHR could take them away from the business of nursing:

*Nurse (Sister): “We’re all a bit scared of is it going to be task oriented, taking you away from your patient care … taking time away from the patient so we can tick all the boxes on the system … ” (10:20-26).*

Senior clinicians, who were not members of the EHR support team, expected to benefit least from implementation, primarily because use was perceived to have the potential to slow down their pace of work:

Doctor (Consultant): “*When I clerk someone … I'm going to have to put that on to* [EHR*]. Takes me two seconds to write it down … It's going … to take me 30 minutes … well, I don't know, 15 minutes a record plus. It's not going to be quick.” (4:160-165).*

A cumulative effect of least benefit existed between senior clinicians and outpatient services. The relatively fast pace of patient flow in clinics, and a perception that the staff working in these services were less computer literate than their acute services colleagues, meant that the introduction of the EHR was perceived as a potential threat to service delivery: *Doctor (Consultant):* “… *I think outpatients will be an absolute disaster...” (4:516).*

### Cognitive participation – are staff prepared to engage and commit to using the EHR?

All participants viewed the EHR as central to delivering patient care, and were motivated to invest in implementation. Participants with previous experience of using an EHR (mainly junior doctors and members of the EHR support team) were relatively confident in the benefits to be derived from change in their usual practice: *Doctor:* “*… the consistency in care with things that we miss out quite often will obviously be a big benefit.” (2:152–154).* For other participants (mainly senior clinicians and nurses), they were concerned that they were ill-prepared to use the EHR. Their concerns were based around four main issues: lack of consultation/preparation for context-specific needs and wants, equipment and usability, formal training and support for introduction of use.

### Concerns raised about using EHR

The perceptions that some participants held about the way in which the implementation programme had been enacted impacted negatively on their engagement with the EHR. The Trust had put in place strategic planning for uploading data into the EHR, yet several participants lacked knowledge of these and were anxious that ultimately front-line staff would be required to complete the majority of this work:

*Nurse: “ … we don’t have any ward clerks … we have to wait for admissions to do it … so we’re waiting to put a patient actually on to the system before we can do anything really … ” (13:351-357).*

Despite a positive appraisal for the perceived benefits of the EHR, some health professionals felt unprepared to operationalise the system within their usual work practice. Senior staff reported a lack of engagement with them as to how the EPR could best work for them:

*Doctor (Consultant): “No one’s engaged with us at what we want on the wards and we are being told what we want” (4:44-46).*

Participants were concerned that patients with complex needs or co-morbidities did not easily fit into EHR templates. They were concerned that drop-down menu options would be rigid, which could result in triggers for tests, which, in clinical opinion, may not be necessary:

*Doctor (Consultant): “ … One of the problems with my particular speciality … is that everybody has got a slightly different type of problem … if you’re a delirious 80 year old, that can be because you’ve got subdural haematoma; it can be because you’ve got a UTI; it can be that you’ve just got dementia. So it doesn’t fit easy into a tick or drop-down box … and you’ll just have to populate various things, which will then populate various tests … So that concerns me.” (8:51-60).*

Those participants who believed that the go-live weekend was imminent were concerned that they lacked access to computer equipment or lacked physical space in which to operate computers. Additional challenges related to the practicalities of agency staff using the EHR system. For example, for wards that depend on agency staff, there was concern that these staff may not know how to use the system, and that this deficit would lead to an increased workload for nurses. Despite online training provision for agency staff, participants were concerned this pre-requisite would put some agency staff off coming into the hospital, thereby reducing further the numbers of staff available:

*Nurse: “ … we don’t even know how the agencies [staff] are going to log in to it. They just all going to turn up on that night and we don’t have a clue what they’re going to do. Apparently at other trusts they have got to go and get the nurse in charge to verify what she’s doing … ” (11:692-696).*

### Training and support

To support staff commitment and engagement with EHR, the Trust provided mandatory training events and additional resources including play domains (simulations of the EHR, which allow staff to practice using the system), and super-users (a group of health professionals that received additional training on the system). Participants were divided as to the impact of their engagement with training on their expectations of the EHR system. Junior doctors were relatively confident in their skills and abilities to use the EHR, with one junior doctor reporting that they had treated formal training sessions as an opportunity to ask questions that they had generated through using the EHR play domains. However, others felt they had not received enough training, or found it too intense or generic:

*Nurse: “We are not trained enough to be sure we know what to do … I don’t feel confident to back up somebody who doesn’t know what they are doing.” (11:218-224).*

Many participants were not experienced in using computers in their daily work practice and reported a lack of opportunity to move beyond the classroom setting. Some participants believed that the training inadequately addressed generational differences in computer literacy and felt that it fell short of their expectations:

*Doctor (Consultant): “ … the people who did the education just told us what they wanted us to know. They didn’t work out what I needed to know to make it work” (4:331-333).*

There was dissonance between staff expectations and training objectives. One senior member of staff suggested that: “*… the knowledge of the system is now ready, the skill of how you use it will only happen when we go live …*” *(Manager, 7:63–65).* However, lack of capacity during shift hours and lack of access to play domains impeded some participants’ ability to engage with the EHR. Where they were able to practice on play domains, some participants found there was inadequate simulation of what they would do in practice:

*Doctor (Consultant): “The play domain isn’t fit for purpose, for a number of reasons … it isn’t integrated as it should be … ” (8:77/120)*, and:

*Nurse: “ … some of the patients don’t have drug charts set up on them, and yet it’s a nurse domain but nurses don’t prescribe. So that part of the training package is not quite really what it should be … ” (1:175-177).*

Several participants reported that they were efficient in performing ‘little tasks’ using the EHR, yet were anxious as to how they would integrate use of the EHR into their usual working practices:

*Doctor (Consultant): “ … There’s a lot of stuff in the middle, which is the important bit … and that is why so many people are anxious about what is going to happen in three weeks’ time” (8:154-158).*

This was compounded by uncertainty over the level of support that would be available to them, particularly during the early implementation phase. Some participants were suspicious that plans for additional resources would not materialise, and they would be pushed to deliberately fail in order to gain access to additional support:

*Nurse: “ … I think we have to fail in a way in order to … get loads of screens in there.” (11-12:416-422).*

### Collective action – do staff feel able to do the ‘work’ to use EHR?

All participants believed that they had completed the official training programme, and had, to varying degrees, engaged with the additional resources that were available to them. The extent to which they perceived that this had prepared them for EHR implementation was influenced by perceived compatibility of the EHR with existing work practices. Similar to findings reported above, the perceptions expressed by junior doctors indicated that they were least concerned about the impact of the EHR on their working practice. Other participants reported concerns for perceived changes in their working relationships, patient flow and available information which may impact their ability to do the work of using the EHR to improve patient care. However, participants were unanimous that they would have to find ways to make the EHR ‘work’ for them in practice:

*Nurse: “ … we have in our practice found out that you don’t have to fill them all out, so we’re already cutting corners.” (1:545-546).*

### Working relationships

The role of junior doctors was expected to respond to and evolve with EHR implementation. The dynamic of ward rounds was perceived to change from consultants documenting clinical decisions to junior doctors having a more active role in care plans. Junior doctors expected to be doing most of the documentation, most of the time, which led to some concern that they would become clerks for their consultants and result in missed learning opportunities:

*Doctor (Consultant) “ … one of my issues with junior doctors is that they will spend time being clerks on the computer rather than being a junior doctor … they won’t be behind the curtain with [the patient] … I think it will have a significant impact on their potential training on the job”. (8:512/551-555).*

It was anticipated that some members of staff would require more support to use the EHR than others. With the introduction of the EHR, some participants were concerned that junior doctors would be left to: *“sort their own selves out … and get themselves up to a certain level” (Nurse, 1:94–95).* There was variation in understanding of the anticipated change to working relationships between different professions, with some staff unclear as to how their role would evolve: W*ard Clerk:* “*… but apparently there are other things that we’re going to be doing instead [of filing paper records], which I don’t know …*” *(5:116–117).* Unfortunately, some participants anticipated that staff may leave the NHS as a result of implementation as they would find use of the EHR too cumbersome:

*Nurse: “ … some staff on the ward are older and are frightened of the computer, even in this day and age. Two staff may leave on the back of this, because I think they will find it too much … ” (1:27-30).*

One participant suggested that where there was strong team cohesion, they were confident that they would ‘ride the storm’:

*Nurse: “ … we’re a good team on here, and I think if they can’t manage on here then they’re not going to manage anywhere else; and we know that it’s doable … ” (1:105-107).*

### Patient flow

With the introduction of the EHR, consultations, including ward rounds, were expected to take more time to complete. Usual practice on in-patient wards is for junior doctors to complete lists of tasks for different patients after the daily ward round. However, EHR use would require staff to complete tasks such as recording allergies, ordering tests, and prescribing medications during the ward round, which was expected to increase their duration and alter the dynamic:

*Doctor: “ … typing it all out, and drop down boxes, and searching … which is just a long drawn out version of what we do at the moment. So it will take longer … ” (2:200-206).*

Participants accepted that compulsory completion of templates may reduce the risk of important information or decisions being missed. However, anecdotal reports from a neighbouring hospital who recently implemented the same electronic system caused concern. Specifically participants discussed the potential for the EHR to increase duration of ward rounds, which may delay discharges, affecting A&E waiting times and in turn pose risks to patient safety. They also based their perceptions on experiences in primary care following the introduction of EHR. Participants were also concerned that sometime after implementation in primary care, wait times had not returned to pre-implementation levels:

*Doctor (Registrar): “You go back to GPs … When their electronic records came in years ago they were on six and two third minute appointments. They changed to ten minute appointments and they’ve never been able to go back … ” (14:120-123).*

Similarly, in out-patient services, participants were concerned that EHR use would limit and slow down productivity in services which were ‘*working flat-out’ (Consultant, 4:66).* Longer wait times as the staff got used to using the EHR system were anticipated-with services considered unprepared to respond. Although there was a planned 25% reduction in clinic referrals for the first 2 weeks of the EHR going live, some participants believed that this did not allow enough time for the system to be fully embedded. This was compounded by an observation that the majority of staff working in out-patients were comparatively slow typists and so EHR implementation was, to a point, considered an unjustified additional use of time. As a result, the initial implementation period was predicted to be:

*Doctor (Consultant): “ … horrendous … ” (9:326)* and *“ … there’s no turning back now, it’s going to happen … we wait with baited breath” (8:820/828).*

### Available information

Implementation of the EHR required changes to be made to the nature and type of information that could be recorded. This was perceived to be particularly complex for nurses who record lots of different types of information from different sources. Participants were concerned that important information that could impact patient care would be lost, due to the sheer volume of information that nurses acquire and are required to record:

*Nurse: “ … you could take a phone call from some relatives who were concerned about their mum, and you could be on the phone for 45 minutes, and you are getting all sorts of information thrown at you … you could have 4 or 5 of these conversations in one day … Most of us are only two-finger or one-finger typists … We’re worried about how long it is going to take up to record accurately their concerns … so that nothing gets missed.” (1:419-430).*

Similarly, clinicians were concerned that they would not be able to provide a comprehensive picture of their thinking around patient care, which may change the nature in which clinical opinion is communicated. Some participants were worried that although they could find ways to work around this issue, the rationale underpinning their clinical decisions would be lost through use of the EHR. The loss of information on clinical opinion was considered to potentially result in a lack of transparency as to how patient care is carried out:

*Doctor (Registrar): “You will lose a lot of information … you really need all that information in there … because it is a clear record of what story we were given, what examination findings we were given and what is the clinical opinion. And that is still a really vital part of what we do … there is a danger of losing some of that information … ” (14:166-171).*

### Reflexive monitoring – how staff appraise the EHR

Participants appraised the EHR by identifying a number of advantages and disadvantages to using the system.

### Advantages

All participants perceived long-term benefits, which coincided with the official perspective on EHR implementation to the need for improved: accessibility and availability of records, efficiency, research and communication with other health and care organisations. The potential for future benefits promoted engagement with the EHR:

*Doctor (Consultant): “I think once they first start out, there’s going to be a lot of input going in. But the benefit after a few years is when they [patient] come back to us, you’ve got all the history, you’ve got all the past medical history, you’ve got the drugs, instantly you can see what they’ve been in for before … there’s no delay … ” (9:288-293).*

Participants believed that patient safety and quality of care would be improved through use of the system. They expected that EHR use would result in a reduction in risk of errors, particularly around prescribing. They also anticipated transparency in errors and safer practice as all information would be legible and collected in a consistent manner:

*Doctor: “ … I think with prescriptions and prescribing, often it [EHR] flags up errors. So I am hoping that if … you try to prescribe … five hundred grams of amoxicillin which I have seen … it will flag that up and say, that is not an appropriate dose for a drug … ” (2:158-163).*

### Disadvantages

Some participants (those not involved in EHR set-up) were concerned that the potential for intelligent problem solving was missing. There was a tension between standardisation and localisation of the system. Users (clinicians) could not communicate with software developers directly and they believed that the *EHR friends*, who were mainly administrative staff, could not enhance the system directly. The individual needs of specific specialties, and a perceived complex chain of command in making changes to resolve such issues and the way in which the system could be customised was not transparent:

*Doctor (Consultant): “ … the people telling you how to do it are telling you how they think you should do it and not telling you how you currently work, and therefore how the system will best be developed for you … ” (4:58-62), and Doctor (Registrar): My understanding is there are going to be people about. There are EPR friends. I don’t know any... I’m just going to wait and see and deal with what we’ve got and take it from there” (14:365-368).*

For some participants, there was uncertainty as to what actions they could take if the system was not working for them, with the exception of reverting to paper records:

*Doctor (Registrar): “ … We’ve got to maintain patient safety … I’m going to have a sheet of paper that I will … I’m sitting in front of the patient, I’m still going to have my little notes … So whether they want to keep that bit of paper as a record for whatever reason, I’m going to leave it for them to decide … ” (14:371/482-488).*

Use of the EHR was expected to expose further frustrations in the hospital system and that blame could falsely be apportioned to the EHR. Participants were also concerned that patients could be harmed as people did not know how to use the system. To off-set this, EHR implementation was ultimately perceived as moving towards ‘paper-light’ as opposed to a paper-less system:

*Manager: “I am anxious that we’ll harm patients because people don’t know how to use system, haven’t got the skill. But the mitigation to that is that the patient takes priority, the system is just there. If you can’t get it to work or you don’t know how to do it, you write on a piece of paper … ” (7:265-269).*

## Discussion

This study explored health professionals’ expectations of EHR implementation. NPT provided a framework that has characterised a range of factors that staff perceive to impact their understanding of the purpose of (coherence), engagement with (cognitive participation), anticipated use (collective action) and appraisal of (reflexive monitoring) their preparedness for the EHR.

Our study identified that health professionals perceive potential value in using EHR, and that benefits of use would be reaped in the future. They were willing to engage and commit to EHR use, but for some staff the opportunities for them to do so were limited. There was variation in health professionals’ perceptions on their ability to do the work required for successful implementation. Despite most staff believing that EHR implementation was imminent, there were still challenges with acceptance.

The most obvious finding to emerge from the analysis was variation in preparedness for change at an individual, professional and system level. Opinions differed as to the anticipated impact of EHR use on roles, relationships and interactions. A variety of perspectives were expressed about sufficiency of training and support in preparing staff to be able to do the work. A recurrent theme in the interviews was that acute services and junior doctors were perceived to be the main benefactors of an EHR. This manifested as services which could be responsive to the system. Outpatients services, nurses and those senior doctors who were not involved in the implementation support team, would have to react to the system. This finding is consistent with post-implementation literature that has identified barriers and facilitators that are specific to professional and individual priorities [28, 66] and suggests that electronic records are often viewed as a set of clinical systems for primarily clinical users [11].

In their accounts of perceived impact of use of an EHR on ability to do their work, some staff anticipated reduced performance with the introduction of EHR. Previous literature has highlighted that early users of EHR systems experience a performance dip as they struggle with an unfamiliar system [67]. Common negative impacts reported include changes to workflow and work disruption [1]. The disruption to workflow and changes required are significant challenges for users, particularly in systems that have limited modularity and configurability [68]. In the past, failure to implement has occurred because health professionals found that the systems did not meet their needs and required work-arounds in order to complete work procedures [69]. Electronic transmission of referrals, requests and reports for example were reported as making some workflows faster overall. However, individual stages of these workflows could become more or less time-consuming than the work system that was previously operational, with a range of consequences for the different staff involved [11]. There is a growing body of evidence that a new technology is easier to embed if there is a mutually supporting relationship between technical, social and organisational factors in which new, often unanticipated ways of working are allowed to emerge [70]. Some workarounds may, in some cases, result in more efficient ways of working [70]. Whilst it is beyond the scope of the current study to explore the extent to which certain workarounds were encouraged by the organisation, it can be assumed that concerns about reduced performance that are reported here are potentially preventable through a perceived capacity for more effective design and tailoring of the EHR to meet local requirements. Further work is required to establish why and how preparation to use EHR worked well for some staff and professional groups, and not others.

Another important finding was that nurses were concerned as to how they would record the volumes of information that they usually record. There is an emerging, but limited evidence base that has reported on the negative impact of EHR implementation on nursing practice when documenting crucial patient information [27, 71–73]. Nurses have in the past had minimal influence in the design of systems [71]. The perceived resistance to EHR has been explained as defying poorly designed systems that fail to meet the needs of documentation of nursing practice [73]. There is at present a lack of studies that explore nurses’ experiences of EHR in hospitals, and work is needed to understand the specific needs of nurses with regards to using EHR. Surprisingly, there is also a lack of literature on the accounts of senior clinicians (who were not involved in developing implementation strategies) who are responsible for specific services within hospitals. The findings reported here have also drawn attention to the potential loss of information usually recorded in the clinical decision making process of patient care. There is at present no literature that reports on how this perceived loss impacts patient care. One possible explanation for these findings is “a lack of correspondence between the design of technological properties and the culture of professionals” ( [74], p.221), and adds to the growing literature advocating a cultural approach to the study of technology in organisations [74, 75]. This suggests that implementation of EHR could be improved through efforts to ensure that professional group and service context are much more visible within the implementation preparation plan. The findings also indicate that the traditional roles of some health professional groups could be altered, significantly, through introducing organisation-wide EHRs. Further research is needed to understand the nature of any change to traditional roles and how these changes can be translated into factors associated with engagement.

Despite concerns, a common view amongst staff was that they were confident that they would find ways to make the system work for them. A recurrent theme within their narratives was that more could have been done to support them so as to make the transition to EHR less onerous. Our study has identified a perceived lack of user involvement in preparing for implementation. In the past, EHR implementation programmes have been criticised for being too centralised, for not engaging with healthcare organisations and their healthcare professionals, and for flawed procurement processes [13]. Failure is often linked to implementations where healthcare professionals perceived that they were not involved or listened to about specific requirements. Conversely, a high degree of user involvement is associated with successful implementation [76, 77]. The use of pilot testing phases has been linked with successful implementation, where user feedback on their requirements informed the implementation strategy [78]. In a rare discussion on successful implementation we reported on a bottom-up, user led development of an EHR at a different NHS hospital [79]. The EHR was developed by an in-house team and evolved over an extended period (at a fraction of the cost of commercial EHRs) and illustrated the value of user involvement in EHR design. A positive feedback loop in which users were listened and responded to supported development and further growth of the system. Characteristics of success were high levels of user activity by large numbers of diverse users who reported that they were getting significant benefits from its use.

**Fig. 1:**
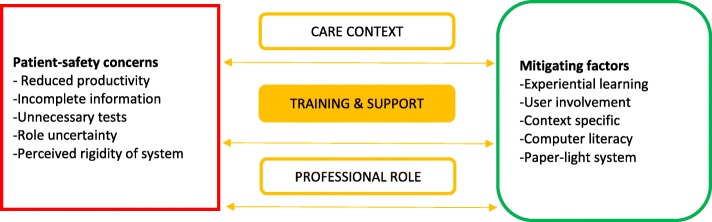
Health professionals’ perceptions on factors that influence successful implementation of EHR

In our study, some staff were concerned that use would cause risks to patient safety. The narratives of staff suggested that their level of preparedness mitigates patient-safety concerns. The relationship between staff narratives on their preparation for use and patient safety concerns is depicted in Fig. 1: Health professionals’ perceptions on factors that influence successful implementation of EHR. In the period preparing for the hospital-wide EHR implementation health professionals’ main concern was the impact on their ability to provide safe care for patients. They expected that implementation would have potential benefits and identified events and actions that could mitigate potential risks to providing safe, quality care. Perceptions varied across different staff groups and specific care contexts with regards to how their training and perceived levels of support impacted patient safety.

There is growing interest in the relationship between EHR and patient safety within the literature [14, 30, 80–84]. As the adoption of EHRs matures it will be possible to further explore this impact to patient safety. A recent analysis of patient safety incidents in fully digital hospitals shows that human-computer interaction is associated with most health information technology incidents [85]. Nevertheless, there exists a perception that there are associated risks with implementation, most notably through incomplete or unavailable information [9, 82], which warrants further investigation. The findings from our study draw attention to the way in which an EHR potentially makes incidents that threaten patient safety more visible. Implementation of EHR systems may also change the nature of risk and introduce new failure modes/incident types. Work is required to understand how EHR use influences detection of and management of patient safety concerns.

### Strengths and limitations

A key component of our analysis was understanding how contextual and human factors influence preparedness for EHR use within the case analysed and as such generalisation to other contexts and programmes should be undertaken with caution. However, the findings build on existing evidence of EHR implementation and map onto the constructs of NPT – a recognised theory for understanding change – and may be considered transferable to other hospitals implementing EHRs. The two-staged approach to analysis and the quality checks on the coding frameworks that were developed during the analysis ensured rigour in the research process. The small study sample and recruitment method may potentially have only included participants who were directly invested in successful implementation and those that had strong concerns about EHR. However, the purposive sampling frame and decision to undertake data collection across four services, which broadly represented key areas of hospital activity, ensured that we obtained diversity in clinical setting, professional group and grade.

NPT provided a useful set of conceptual tools to aid understanding of preparing for EHR use as a dynamic process. Using NPT enabled insights to be gathered on the ‘work’ that is involved in implementation [50]. In their recent review of the use of NPT in implementation research, May and colleagues identified over 100 studies that demonstrated that NPT can effectively assist in explaining success and failures of specific implementation projects [50]. Previous studies have reported on the work that people do during the adoption process. The way in which NPT was used in this study adds to this body of knowledge through showing how it can facilitate systematic access to users’ perceptions which can then be translated into a useable format to develop interventions that may be required prior to EHR implementation. However, as acknowledged by May and colleagues, NPT places undue emphasis of individual and collective agency without explicitly locating this within, and as shaped by, the organisational and relational context in which implementation occurs [50]. Additionally, we do not know the extent to which some mechanisms are more important than others in determining implementation process outcome. The broader processes of sociotechnical change, such as that defined in the recently developed non-adoption, abandonment, and challenges to the scale-up, spread and sustainability (NASSS) technology implementation framework for predicting and evaluating the success of technology-supported interventions [43], are beyond the scope of the present study. However, the generative mechanisms characterised by NPT are examples of self-organising mechanisms in complex adaptive social systems [37], and as such are useful tools for exploring the dynamics of human agency in implementation.

### Recommendations

A clearer understanding as to how staff groups and services differentially interact with an EHR as they go about their daily work is required. This should include adaptation of the system to reflect this understanding. Our findings indicate that more opportunities for nurses and senior clinicians to engage in preparation for use are needed. Research that measures preparedness for change and factors that mitigate common and unique challenges to implementation should be prioritised. The methods through which staff find ways to make EHR work for them in practice need to be better understood. The heterogeneity of implementation programmes poses challenges for synthesising evidence for successful implementation. Detailed case studies are the cornerstone for understanding how technologies get embedded into healthcare and longitudinal studies that investigate sustainability and scaling up, and that focus on implementation processes, are required [37]. The NPT framework offers the potential to explore local contextual factors, or normal conditions of practice, and to compare implementation elements across different settings.

## Conclusions

Hospital staff were motivated to invest in EHR implementation and perceived strong benefits to use, that would be realised, after an initial embedding period. Perceptions varied across different staff groups and specific care contexts with regards to how training and support impacted their preparedness for EHR use. This variation suggested that some staff would be responsive to the system, whereas others would be reactive. These differences were related to the perceptions that staff held around their opportunities to engage in preparation. The four core mechanisms of NPT provided a useful framework to explore individual and group expectations for change and outcome following procurement of an EHR. Given the difficulties often seen in implementation, and the political pressure to move forward with the universal adoption of EHRs, more research is needed not only on the effectiveness of EHR, but importantly, on what can be done to facilitate the implementation of EHR.

### Key learning points

- Health professionals’ perceived potential value in using EHR and that benefits to use would be reaped after an initial embedding period.
- Health professionals were motivated to invest in implementation.
- There was variation across staff groups and services on the perceived impact of EHR use on their ability to carry out their role.
- Junior doctors and acute services could be responsive to the system. Outpatient services, nurses and senior clinicians would have to react to the system.
- There was variation across staff roles and services in perceived opportunities to facilitate their engagement. Nurses and senior clinicians perceived that they were least prepared, and that opportunities for them to engage in preparation were limited.
- There was consensus that staff would find ways to make EHR work for them in practice and that this would likely involve a move towards being a paper-light, rather than a paper-less system.
- The four core mechanisms of NPT provided a useful framework to explore individual and group expectations for change and outcome following procurement of an EHR.

## Supplementary information

**Additional file 1.** Interview topic guide.

**Additional file 2.** Coding framework showing main themes, sub-themes and illustrative quotations.

## Data Availability

The data-set which we have acquired will not be shared as a supplementary file. Our ethical approval does not permit the sharing of the entire dataset which we have acquired. Additional file 2 provides an overview of the coding framework from the analysis of interview transcripts.

## Abbreviations

EHR: Electronic Health Records
EPR: Electronic Patient Records
NASSS: non-adoption, abandonment and challenges to the scale-up, spread and sustainability framework
NHS: National Health Service
NPfIT: National Programme for Information Technology in the NHS
NPT: Normalisation Process Theory
UK: United Kingdom
