# Supplementary material for: Staff expectations for the implementation of an electronic health record system: a qualitative study using normalisation process theory
Source: BMC Med Inform Decis Mak. 2019 Nov 14;19:222. doi: 10.1186/s12911-019-0952-3 (PMC6854727; doi:10.1186/s12911-019-0952-3)
Supplement: Supplementary file 1 — Additional file 1. Interview topic guide. [file 12911_2019_952_MOESM1_ESM.pdf]

Additional file 1: Interview topic guide

Background information: How long have you been in your specialty, for how many years have you been working at the trust?

Coherence:

- What do you think an electronic patient record is and what does it do?
- How do you think the electronic record differs from the paper record?
- What do you think the benefits of the electronic record will be and for who?
- What do you think the reasons for introducing the system are?
    - Were you made aware of this formally (e.g. by the trust) and if so how (e.g. formal events promoting engagement).
- How do you think the electronic record fits in with the overall aims/ambitions of the trust?

Cognitive participation:

- Do you have any concerns about the system?
- Do you think the system is a good idea?
- Has the trust provided any training/support or events explaining how to use or about the introduction of the electronic record?
- Are there any staff dedicated to helping the implementation/use of the system?
- Have you been given any dedicated time to get used to using the new system or attend on-going training?

Collective action:

- How do you think the electronic patient record impacted your practice?
    - Will it help or impede you?
- To what extent will you still using paper?
- How will the electronic record fit in with existing work practices (e.g. ward rounds, consultations)
- How do you think the electronic patient record will impact on the working relationships between and within wards/specialties?

Reflexive Monitoring:

- What do you think will be the long term benefits of the electronic record?
- What barriers do you think there will be to using the system?
- What disadvantages do you think there will be to using the system?
- What do you think will be the impact of the system on patient safety?
- How do you think patients will respond to the electronic system?
- Will there be opportunity for users to feedback or adapt/improve the electronic record?

End the interview:

- Thank participant ask if they have any other comments
- Explain again about how data will be used and reiterate about anonymity and confidentiality
- Provide opportunity for questions and states that the chief investigator is contactable after the interview should questions arise.
