# Supplementary material for: Staff expectations for the implementation of an electronic health record system: a qualitative study using normalisation process theory
Source: BMC Med Inform Decis Mak. 2019 Nov 14;19:222. doi: 10.1186/s12911-019-0952-3 (PMC6854727; doi:10.1186/s12911-019-0952-3)
Supplement: Supplementary file 2 — Additional file 2. Coding framework showing main themes, sub-themes and illustrative quotations. [file 12911_2019_952_MOESM2_ESM.pdf]

Additional file_2

Main themes, sub-themes and illustrative quotes of the theme **Coherence**

Main theme	*Sub-theme*	*Illustrative quotations*
Understanding of reasons for introduction	Digital age	"…but it's quite obvious to me that eventually everything is going to have to be electronic, because this is where the world is going…" (Doctor, 2:323-325)
Purpose of EHR	Different purpose dependent on staff role	"…because it's going to get rid of a heck of a lot of paperwork for them [clinicians], and everything's going to be there for them to look at" (Ward Clerk, 5:83)
Anticipated benefits	Value in implementing EHR	"We will be in a situation where that information will be instantly available every time, whereas at the moment you have to go back through the case record…" (Manager, 7:58-60)
Who think will benefit	Different benefits for different staff roles	"…we can type a lot quicker than we can write, the juniors anyway…" (Doctor, 2:169-170)
How it differs or compares to paper records	Amount of information is overwhelming	"…it's overwhelming; I suppose it's the overwhelming amount of information that you see on that screen that people think, oh my god, I don't know where to go…" (Nurse, 1:537-539)
	More time consuming	"So we are going to have to go in and click stuff. It takes me two seconds to write a CT card. Now it will take a minute, every card…" (Consultant, 4:148-149)
	Concern over receiving timely information	"…the bit that really, really worries me is that if a test result or something comes up, I'm not convinced that I am going to get it…the ward staff just flick through the notes, they recognise my writing in the notes…" (Consultant, 8:279-283)
	Different information collected in EHR	"…some of the criteria on there that we would normally fill out aren't on the [EHR] template…you are still going to be wanting to write that information down, because it is important" (Sister, 10:235-239)

Main themes, sub-themes and illustrative quotes of the theme **Cognitive participation**

Main theme	*Sub-theme*	*Illustrative quotations*
Concerns that have about using the system	Infrastructure not in place	“I am concerned that the infrastructure will not be adequate. I see no evidence that the infrastructure has been put in place currently with a month to go or whatever for us to deliver this.” (Consultant, 4:43-45)
	Not tailored to specific needs	“The people who are training, to me, needed to come and see how the wards work before they did these courses, because every ward is different." (Ward Clerk, 5:67-69)
	Fitting patients’ needs into the system	“…a lot of it is the time in finding the bit to tick, because it’s all ticks and clicking onto stuff. And do we really want to know whether somebody has a gun and it’s locked up at home? Well, I’m not going to be asking those questions; why are they there?” (Nurse, 1:516-520)
	Trigger unnecessary tests	“…you’ll just populate various things, which will then populate various tests, and all this kind of stuff. So that concerns me.” (Consultant, 8:59-61)
	Access to equipment	“…and we’re just worried there’s not going to be enough equipment per person and then we’ll just be…queuing…” (Nurse, 12:16-18)
	May miss clinical work	“…taking time away from the patient so we can tick all the boxes on the system, as such.” (Sister, 10:26-27)
	Slow everything down as takes time to use equipment	“…I think it’s going to take time to recognise what the changes are. I think it’s going to slow us down; I think it affects, initially, flow…” (Consultant, 8:11-12)
	Loss of important information	“You’ll lose it...lose a lot of information. Now some people would argue that you really need…all that information in there...and that’s still a really vital part of what we do…Some information will get lost I imagine potentially.” (Registrar, 14:166-175)
	Agency staff reliance	“…we’ll have agency…the thing is if we’re really short staffed, which…and it’s…like I say, loads of people coming in and the agency nurses can’t do anything…” (Nurse, 12:724-736)

Main themes, sub-themes and illustrative quotes of the theme **Cognitive participation (continued)**

Main theme	*Sub-theme*	*Illustrative quotations*
Training and support	Learning from experiences of others	"...I would have liked to have gone...because I know [neighbouring trust] have been on it for about 12 years, and I would have liked to have gone there just to see how it works, because I know they've had a lot...just hearing on the grapevine, they've had a lot of proper teething problems." (Ward Clerk, 5:215-2185)
	Not enough training	"You should have…like, to me, you should have two weeks intense training or something like that, you know, when you're…or at least week." (Nurse, 12:790-791)
	Training not tailored to individual needs	"I've done my training. I've done me…done the training and what have you...absolutely upfront with you, I've forgotten it." (Registrar, 14:330-331)
	Concerns not heard	"EPR trainers need to know what I do … and how the ward works; it's slightly different to how that ward works. So in a sense, they should be out now for them trying to understand what are the nuances that we may get here." (Consultant, 8:257-260)
	Unable to access training	"Staff have been going and they're got there and the training has been cancelled, or the system is down, that's been nearly every week. Somebody got cancelled yesterday, found out at the last minute it was going to be cancelled for today because somebody was off sick." (Nurse, 1:145-158)
	Inadequate simulation of daily practice	"...here's a play domain that we can go into, it's not what we thought it was going to be, some of the things have not been set up properly, so there's a lot of things on there that we don't need to see, and some of the patients don't have drug charts set up on them, and yet it's a nurse domain but the nurses don't prescribe. So that that side of the training package is not quite really what it should be, I don't think… (Nurse 1, 171-176)
	Uncertainty around on-going support	"…but the EPR friends aren't necessarily people who know what I do." (Consultant, 8:237-238)
	Uncertainty about availability of additional resources to support implementation	"Well I was with matron when I said, there are a lot of floor walkers coming. She said, oh I wouldn't be so sure about that. That was her response the other day, so…yeah, so I don't know. (Nurse, 11:279-281)

Main themes, sub-themes and illustrative quotes of the theme **Collective action**

Main theme	*Sub-theme*	*Illustrative quotations*
Perceived impact on practice	Work more seamlessly	"I think ultimately it will be better, because it should work more seamlessly." (Consultant, 4:20-21)
	Improve workflow	"Potentially, if the junior doctors are really slick they will be able to do it there and then, all the tests and things fairly quickly…" (Registrar, 14:421-423)
	Change nature of relationship with patients	"…it could disrupt that intimate relationship with that patient when they are being clerked in or on the ward round for example…" (Doctor, 2:261-263)
Perceived impact on existing work practices	Slow productivity	"The ward rounds just take longer and jobs that were getting done on the ward rounds aren't getting done on the ward rounds, and they're getting done after the ward rounds. So, that's for drug charts and that sort of thing. So, people just getting...their discharges are getting delayed because of that, so they said".(Consultant, 3:29-133)
	Change in nature and type of information flow	"…there's no hiding in the system, you know, you...it's all, and it's those little work arounds that you've had, little fudges that you've done with sort of processes really." (Consultant, 3:333-335)
	Perceived impact on use of paper records	"Realistically, we probably will (continue to use paper) because I can't imagine any of it going that smoothly. So, for example NEWS charts, I think…you know, I can't imagine it's going to go straight away just in the NEWS chart, bin the paper version and go straight on to EPR, we'll also have those." (Doctor, 2:69-72)
Perceived impact on working relationships	Increase dependency on other services	"We're not a surgical ward on here, and we were shown how to send someone to theatre; it might happen twice a month on here, so I think we're going to have to call probably a surgical ward to help us through that because you're not going to do it enough even to remember, I don't think so anyway, and people are going to say they can't remember what to do, because there is so much." (Nurse, 1:62-67)
	Increase support required for less computer-literature staff	"I'm not someone who has been brought up using computers. I'm not…the job is not desk-based". (Consultant, 4:42-43)
	Increase compromise between staff working	"It's what equipment we've got available to us?..Because it's…a bit of give and take between yourselves and the other members of staff…" (Nurse 14:402-403)
	Improve communication between specialties	"So with the EPR, it's all computer based, and everyone's going to be able to read it easily, so that's the most important thing for me, personally. I mean I'll be able to read documents and consultant's reports clearly, so...and it's quicker." (Doctor, 6:130-133)

Main themes, sub-themes and illustrative quotes of the theme **Reflexive monitoring**

Main theme	*Sub-theme*	*Illustrative quotations*
Perceived long-term benefits	Improve efficiency	"So I think there will be a lot of benefits, but I don't think we'll realise the benefits for some time". (Consultant, 3:340-341)
	Improve access to information	"I think it's...personally it's a...it should be a better way, a more efficient way, to transfer information between different people." (Doctor, 6:93-95)
Perceived opportunities to adapt system	Uncertain that can influence change	"But they're all options that you have to find your way through; why have they not been removed? But we've been told some things you can remove and personalise it, so we're hoping that the person who is allocated to our ward will come and try and personalise things a bit more." (Nurse, 1:511-514)
	Lack of engagement with individual wants	"We tried to engage with them and I've just been…everyone's now too busy, because oh, we've got to do this and we've got to do this, and no one's coming to talk to you because they've got this deadline of four/five weeks down the line and they're doing it the way they want to do it rather than the way we think we might be able to use it." Consultant, 4:48-53
Perceived barriers to use	Challenge for some groups of staff	"Maybe my division has not engaged very well with it, because we're all a bit Luddites and this, that and the other, or we just don't want anything to change because we're happy with how things work." (Consultant, 4:432-434)
Disadvantages to use	Expose further frustrations in the system	"And I think what it's done is certainly given me a better understanding of what the system is capable of, because the anxiety most people had is it's going to transform their lives, it's going to change everything that they do for the better, and all those frustrations they've had in their workplace are going to be resolved. Actually it's only going to scratch the surface of those frustrations and actually it will expose those frustrations, so it will expose more issues I think going forward." (Manager, 7:33-39)
